# Dynamics of water conveying zinc oxide through divergent-convergent channels with the effect of nanoparticles shape when Joule dissipation are significant

**DOI:** 10.1371/journal.pone.0245208

**Published:** 2021-01-14

**Authors:** Umair Rashid, Azhar Iqbal, Haiyi Liang, Waris Khan, Muhammad Waqar Ashraf

**Affiliations:** 1 CAS Key Laboratory of Mechanical Behavior and Design of Materials, Department of Modern Mechanics, University of Science and Technology of China, Hefei, Anhui, China; 2 Mathematics and Natural Sciences, Prince Mohammad Bin Fahd University, Al Khobar, Saudi Arabia; 3 IAT-Chungu Joint Laboratory for Additive Manufacturing, Anhui Chungu 3D Printing Institute of Intelligent Equipment and Industrial Technology, Wuhu, Anhui, China; 4 Department of Mathematics and Statistics, Hazara University, Mansehra, Khyber Pakhtunkhwa, Pakistan; Central University of Karnataka, INDIA

## Abstract

**Aim of study:**

The shape effects of nanoparticles are very significant in fluid flow and heat transfer. In this paper, we discuss the effects of nanoparticles shape in nanofluid flow between divergent-convergent channels theoretically. In this present study, various shapes of nanoparticles, namely sphere, column and lamina in zinc oxide-water nanofluid are used. The effect of the magnetic field and joule dissipation are also considered.

**Research methodology:**

The system of nonlinear partial differential equations (PDEs) is converted into ordinary differential equations (ODES). The analytical solutions are successfully obtained and compared with numerical solutions. The Homotopy perturbation method and NDsolve method are used to compare analytical and numerical results respectively.

**Conclusion:**

The results show that the lamina shape nanoparticles have higher performance in temperature disturbance and rate of heat transfer as compared to other shapes of nanoparticles.

## 1. Introduction

Nowadays, biomedical nanomaterials have achieved significant attention due to their prominent biomedical applications and biological characteristics. The significant development of nanomaterials and metal oxide nanoparticles displays promising and far-ranging prospects for the biomedical field, especially for anticancer gene/drug delivery, antibacterial, biosensing, cell imaging, etc. Jiang et al. [1] and Mishra et al. [2]. Zinc oxide nanoparticles have very important metal oxide nanoparticles, which are employed in varieties of fields because of their chemical and peculiar physical properties [3, 4]. Zinc oxide has excellent antimicrobial, antibacterial and UV blocking properties. Adding Zinc oxide nanoparticles in finished fabrics in the textile industry has exhibited the most attractive function of visible light and ultraviolet resistance, deodorant and bacteria [5]. Due to strong UV absorption properties, Zinc oxide nanoparticles are also used in care products like cosmetics and sunscreen [6]. Zinc oxide is also utilized in other industry fields involving photocatalysis, concrete production, electrotechnology industries, electronics, etc. [4]. The small size of Zinc oxide makes it more convenient to absorb in the body. Thus, commonly nano Zinc oxide is used in the preservation of food. Zinc oxide nanoparticles have gained much consideration in biomedical applications. Zinc oxide nanoparticles relatively less toxic property as compared to other metal oxide nanoparticles. Zinc oxide nanoparticles have a comparatively inexpensive and excellent exhibit in biomedical applications such as antibacterial, anticancer, diabetes treatments, Drug delivery, anti-inflammation, bioimaging and wound healing [1, 2]. The studies about heat transfer in Zinc oxide nanofluids are relatively rare as compared to other metal oxide nanofluids [7].

Magnetohydrodynamics has various applications in industrial and geophysical fields. In the field of fluid mechanics, magnetohydrodynamics is one attractive topic of researchers. Magnetohydrodynamics is applied in plasma generators, Hall acceleration and cooling of nuclear reactors [8]. Many researchers did work on magnetohydrodynamics fluid flow. Makinde et al. [9] discussed a numerical solution of magnetohydrodynamics Casson fluid flow on an upper horizontal thermally stratified surface. Koriko et al. [10] examined the 3-dimensional flow of water conveying alumina and water conveying alumina Iron oxide with the influence of a magnetic field. Tlili et al. [11] analyzed the 3-dimensional magnetohydrodynamics (AA7072 /AA7075) methanol hybrid nanofluid with slip effect. Ashwinkumar [12] studied heat transfer and mass transfer analysis in unsteady magnetohydrodynamics flow of (aluminum alloy and silver) water nanofluid. Samrat et al. [13] analyzed the effect of the chemical reaction and thermal radiation on the 2-dimensional flow of magnetic nanofluid. Mabood et al. [14] studied the boundary layer analysis of 2-dimensional unsteady hybrid nanofluid flow over a flat slendering surface.

Most physical phenomena and scientific problems occur nonlinearly. To obtain the analytical solution of such problems are partially difficult, excluding in limited problems. Recently, several methods have been achieved significant attention such as the variational iteration method [9], Hirota’s method [10], Homotopy analysis method [11], homogeneous balance method [12] as well as homotopy perturbation method. The first time, “He” introduced the Homotopy perturbation method (HPM) [15]. The Homotopy perturbation method (HPM) is an efficient and powerful technique to find the solution of nonlinear and linear equations. The homotopy perturbation method (HPM) is a combination of the homotopy and perturbation methods. Homotopy perturbation method (HPM) can take significant advantage of the convectional perturbation method while eliminating its restrictions. The homotopy perturbation method (HPM) has been most successfully applied by many authors to solve several types of nonlinear and linear equations in engineering and science [16]. Homotopy perturbation method (HPM) is applied by many researchers to handle varieties of engineering and scientific applications to solve several function equations. The solution of Homotopy perturbation method is supposed as a sum of an infinite series which converges quickly to exact solutions. Many researchers have been conducted their research by applying Homotopy perturbation method (HPM) to the nonlinear and linear equations [17].

Jeffery [18] and Hamel introduced a fluid flow which is known as Jeffery Hamel flow. It is a two-dimensional flow between two parallel walls. Jeffery Hamel flow is caused by a sink or source at the intersection of channel walls. Analysis of Jeffery Hamel flow, mass transfer and heat transfer of fluid between divergent and convergent channels are very important motive [19]. The flow between divergent and convergent is very significant due to its uses in industries, medical, engineering and biomechanics. Various researchers have been tried to extend the flow in converging and diverging channels by considering the impact of different parameters such as slip, magnetohydrodynamic and heat transfer phenomena. Change in angle, it makes a significant role in these flow as discussed in several studies [20]. Sheikholeslami discussed MHD Jeffery Hamel nanofluid flow analytically in the non-parallel wall [21]. Ochieng et al. [22] examined the Jeffery and Hamel flow with nonlinear skin friction and viscosity flow through a divergent conduit in the existence of a magnetic field. Nehad et al. [23] discussed that the Nusselt number proportional to the heat transfer is ascertained at the higher value of suction and stretching ratio the water conveying multiple wall CNT and silicon dioxide.

It is a necessity to find heat transfer under the exact shape of nanoparticles. The shape effect of zinc oxide nanoparticles in Gold-hybrid nanofluid has been discussed by Dinarvand and Rostami [24]. However, the shape effects of zinc oxide nanoparticles in nanofluid water base between non-parallel walls have not been addressed previously. The present study aims to discuss the zinc oxide nanoparticles shapes effects between non-parallel. Furthermore, the results of the Homotopy perturbation method are compared with NDsolve technique and have been reported in the literature to check the reliability and validity of the Homotopy perturbation method.

## 2. Mathematical formulation

In this report, the problem of steady, two-dimension and incompressible nanofluid flow between divergent-convergent channels is considered. Furthermore, it has also considered that flow is uniform along the z-axis and the velocity of nanofluid is radial. It also considers that the velocity of nanofluid is dependent on r and *θ* coordinates. The magmatic field applied transverse to the diction of flow. The equations of the problem are modeled as [25]. Thermophysical properties of zinc oxide (ZnO) and water are presented in Table 1. The values of nanoparticles shape factor are presented in Table 2.

$$\frac{\rho_{n_{f}}}{r}\frac{\partial }{\partial r}(r.v_{r})=0,$$ (1)

$$v_{r}\frac{\partial v_{r}}{\partial r}=-\frac{1}{\rho_{n_{f}}}\frac{\partial p}{\partial r}+v_{n_{f}}\left[\frac{\partial^{2}v_{r}}{\partial r^{2}}+\frac{1}{r}\frac{\partial v_{r}}{\partial r}+\frac{1}{r^{2}}\frac{\partial^{2}v_{r}}{\partial \theta^{2}}-\frac{v_{r}}{r^{2}}-\frac{{\sigma B}^{2}v_{r}}{\rho_{n_{f}}r^{2}}\right],$$ (2)

$$-\frac{1}{\rho_{n_{f}}r}\frac{\partial p}{\partial \theta }+2\frac{v_{n_{f}}}{r^{2}}\frac{\partial v_{r}}{\partial \theta }=0,$$ (3)

$${\left(\rho Cp\right)}_{nf}v_{r}\frac{\partial T}{\partial r}=k_{n_{f}}\left[\frac{1}{r}\frac{\partial }{\partial r}(r\frac{\partial T}{\partial r})+\frac{1}{r^{2}}\frac{\partial^{2}T}{\partial \theta^{2}}\right]+u_{n_{f}}\left[2.({\left(\frac{\partial v_{r}}{\partial r}\right)}^{2}+{\left(\frac{v_{r}}{r}\right)}^{2})+{\left(\frac{1}{r}\frac{\partial v_{r}}{\partial r}\right)}^{2}\right]+\frac{{\sigma B}^{2}v_{r}}{r^{2}},$$ (4)

where *p*, *v*_*r*_, *T*, *B*, *σ* denote the pressure, radial velocity, temperature, electromagnetic induction and fluid conductivity, respectively. Also $u_{n_{f}},v_{n_{f}},\rho_{n_{f}},k_{nf},{Cp}_{nf}$ denote the dynamic viscosity, kinematic viscosity, density, thermal conductivity and heat capacity of nanofluid, respectively.

**Table 1:** Thermophysical properties of zinc oxide(ZnO) and water as [23, 24].

Physical properties	Zinc oxide	Pure water
ρ (kg/m3)	5600	997.1
*Cp* (J/kg K)	495.2	4179
k (W/m K)	13	0.60

**Table 2:** Shape factor parameters of nanoparticles [26].

Shapes	column	sphere	lamina
*ϕ*	0.4710	1	0.1857
m	6.3698	3	16.1576

The boundary conditions of the problem are

$$V_{r}=V_{max},\frac{\partial v_{r}}{\partial \theta }=0,\frac{\partial T}{\partial \theta }=0\;at\;\theta =0$$

$$V_{r}=0,T=T_{w}\;at\;\theta =\alpha$$ (5)

*T*_*w*_ denote the wall temperature.

For radial motion

$$f(\theta )=r.V_{r}$$

The variables are considered as [25]

$$f(\eta )=\frac{f(\theta )}{f_{max}},\;g(\eta )=r^{2}.\frac{T}{T_{w}},\;\eta =\frac{\theta }{\alpha }$$ (6)

where f_max_ denote the central line of momentum. Substituting Eq (6) into Eqs (2–4) and eliminating P, we get

$$f^{\prime \prime \prime \prime }-2Re\alpha [BS]ff^{\prime }+(4-B.M){\alpha^{2}f}^{\prime }=0,$$ (7)

$$g^{\prime \prime }+4\alpha^{2}g+2\alpha^{2}\frac{Y}{H}\mathrm{Pr}\;fg+\frac{PrEc}{ReBH}\left(4\alpha^{2}f^{2}+{f^{\prime }}^{2}\right)+\frac{PrEcM}{H}f^{2}=0$$ (8)

In this study we consider as

$$S=(1-\phi )+\frac{\rho_{s}}{\rho_{f}}\phi ,B=(1-{\phi )}^{2\cdot 5},Y=(1-\phi )+\frac{{\left(\rho Cp\right)}_{s}}{{\left(\rho Cp\right)}_{f}}\phi ,H=\frac{\left[k_{s}+(m-1)k_{f}]-(m-1)\phi (k_{f}-k_{s}\right)}{\left[k_{S}+(m-1)k_{f}]+\phi (k_{f}-k_{s}\right)}$$ (9)

Where *ϕ*, subscript *f* and subscript *s* denote the nanofluid volume fraction, base fluid and solid nanoparticle respectively.

Also

$Re=\frac{af_{max}}{v}$ is Reynolds number, $Pr=\frac{{\left(\rho Cp\right)}_{f}f_{max}}{k_{f}}$ Prandtl number, $Ec=\frac{a^{2}f_{max}^{2}}{{\left(Cp\right)}_{f}T_{w}}$ is Eckert number and $M=\sqrt{\frac{{\sigma B}^{2}}{v_{f}\rho_{f}}}$ is Hartmann number.

The Boundary value conditions of problems are

$$f(0)=1,f^{\prime }(0)=0,f(1)=0,g^{\prime }(0)=0,g(1)=1.$$

The physical quantity Nusselt number is given by

$${r^{2}Nu}_{r}=H.\left[2-\frac{g^{\prime }(1)}{\alpha }\right]$$ (10)

## 3. Homotopy perturbation method

In order to obtain the analytical solution, the homotopy pertutbation method is simulate nonlinear ordinary differential Eqs (7 and 8). The solution of Eqs (7 and 8) by applying the Homotopy perturbation method we conduct as follows:

$$(1-P)(f^{\prime \prime \prime }-f_{0}^{\prime \prime \prime })+P(f^{\prime \prime \prime }-2Re\alpha BSff^{\prime }+(4-BM){\alpha^{2}f}^{\prime })=0$$ (11)

$$(1-P)(g^{\prime \prime }{-g^{\prime \prime }}_{0})+P\left(g^{\prime \prime }+4\alpha^{2}g+2\alpha^{2}\frac{Y}{H}\mathrm{Pr}\;fg+\frac{PrEc}{ReBH}\left(4\alpha^{2}f^{2}+{f^{\prime }}^{2}\right)+\frac{PrEcM}{H}f^{2}\right)=0$$ (12)

We consider f and *g* in the following form

$$f=f_{0}+pf_{1}+p^{2}f_{2}\ldots \ldots \ldots \ldots \ldots \ldots \ldots ..$$ (13)

$$g=g_{0}+pg_{1}+p^{2}g_{2}\ldots \ldots \ldots \ldots \ldots \ldots \ldots .$$ (14)

Assuming $f_{0}^{\prime \prime \prime }=0$ and $g_{0}^{\prime \prime }=0$ substituting *f* and *g* from Eqs (13) and (14) into Eqs (11) and (12) respectively. Also, some arrangement and simplification based on powers of p term we have

$$p^{0}:\;f_{0}^{\prime \prime \prime }=0$$ (15)

$$f_{0}(0)=1,f_{0}^{\prime }(0)=0,f_{0}(1)=0$$ (16)

$$p^{1}:\;f_{1}^{\prime \prime \prime \prime }+2Re\alpha BSf_{0}f_{0}^{\prime }+(-BM+4)\alpha^{2}f_{0}=0$$ (17)

$$f_{1}(0)=0,f_{1}^{\prime }(0)=0,f_{1}(1)=0$$ (18)

$$p^{2}:\;f_{2}^{\prime \prime \prime }+2Re\alpha BSf_{0}f_{1}^{\prime }+2Re\alpha BSf_{1}f_{0}^{\prime }+(-BM+4)\alpha^{2}f_{1}^{\prime }=0$$ (19)

$$f_{2}(0)=0,f_{2}^{\prime }(0)=0,f_{2}(1)=0$$ (20)

$$p^{0}:\;g_{0}^{\prime \prime }=0$$ (21)

$$g_{0}^{\prime }(0)=0,g_{0}(1)=1$$ (22)

$$p^{1}:\;g_{1}^{\prime \prime }+4\alpha^{2}g_{0}+\frac{2\alpha^{2}Yf_{0}Prg_{0}}{H}+\frac{PrEc(4\alpha^{2}{f_{0}}^{2}+{f_{0}^{\prime }}^{2})}{ReHB}+\frac{PrEcM{f_{0}}^{2}}{H}=0$$ (23)

$$g_{1}^{\prime }(0)=0,g_{1}(1)=0$$ (24)

$$p^{2}:\;g_{2}^{\prime \prime }+4\alpha^{2}g_{1}+\frac{2\alpha^{2}Yf_{0}{\mathrm{Pr}\;g}_{1}}{H}+\frac{2\alpha^{2}Yf_{1}{\mathrm{Pr}\;g}_{0}}{H}+\frac{\mathrm{Pr}\;Ec(8\alpha^{2}f_{0}f_{1}+2f_{0}^{\prime }f_{1}^{\prime })}{RHB}+\frac{2\mathrm{Pr}\;EcMf_{0}f_{1}}{H}=0$$ (25)

$$g_{2}^{\prime }(0)=0,g_{2}(1)=0$$

Solving Eqs (15–25) with boundary values conditions we have

$$f_{0}(\eta )=-\eta^{2}+1$$ (26)

$$f_{1}(\eta )=-2\alpha \left(\frac{1}{60}BReS\eta^{6}+\frac{1}{24}\left(\alpha BM-2BReS-4\alpha \right)\eta^{4}\right)+\frac{1}{2}\left(\frac{1}{6}\alpha^{2}BM-\frac{4}{15}\alpha BReS-\frac{2}{3}\alpha^{2}\right)\eta^{2}$$ (27)

$$f_{2}(\eta )=-\frac{1}{30}(A^{2}(\frac{B^{2}{Re}^{2}S^{2}\eta^{10}}{45}+\frac{(36AB^{2}ReSM-72B^{2}{Re}^{2}S^{2}-144ABReS)\eta^{8}}{336})$$

$$+\frac{(10A^{2}B^{2}M^{2}-60AB^{2}ReSM+72B^{2}{Re}^{2}S^{2}-80A^{2}BM+240ABReS+160A^{2})\eta^{6}}{120}$$

$${+\frac{(-5A^{2}B^{2}M^{2}+18AB^{2}ReSM-16B^{2}{Re}^{2}S^{2}+40A^{2}BM-72ABReS-80A^{2})\eta^{4}}{24})}^{2}$$

$$+\frac{(-\frac{1}{120}A^{4}B^{2}M^{2}+\frac{1}{42}A^{3}B^{2}ReSM-\frac{163}{9450}A^{2}B^{2}{Re}^{2}S^{2}+\frac{1}{15}A^{4}BM-\frac{2}{21}A^{3}BReS-\frac{2}{15}A^{4})\eta^{2}}{2}$$ (28)

$$f=-\frac{1}{360}((\eta -1)(360+\frac{4A^{2}B^{2}{Re}^{2}S^{2}\eta^{8}}{15}+\frac{9BR{eA}^{2}((BM-4)A-\frac{242BReS}{135})S\eta^{6}}{7}+((BM-4{)}^{2}A^{3}-\frac{33BReS(BM-4)A^{2}}{7})+$$

$$\frac{514AB^{2}{Re}^{2}S^{2}}{105}+12BReS)A\eta^{4}+\frac{514AB^{2}{Re}^{2}S^{2}}{105}+12BReS)A\eta^{4}$$ (29)

$$g_{0}(\eta )=1$$ (30)

$$g_{1}(\eta )=\frac{1}{ReHB}(Pr(-\frac{1}{30}BEcMRe\eta^{6}+\frac{1}{6}A^{2}BReY\eta^{4}-\frac{2}{15}A^{2}Ec\eta^{6}+\frac{1}{6}BEcMR{e\eta }^{4}-A^{2}BReY\eta^{2}+\frac{2}{3}A^{2}Ec\eta^{4}$$

$$-\frac{1}{2}BEcMRe\eta^{2}-2A^{2}Ec\eta^{2}-\frac{1}{3}\eta^{4}Ec))-2\eta^{2}A^{2}+$$

$$\frac{\left(25A^{2}YPrReB+60A^{2}ReHB+11PrEcMReB+44A^{2}EcPr+10PrEc\right)}{30ReHB}$$ (31)

$$g_{2}(\eta )=\frac{1}{30H^{2}ReB}(A(-\frac{1}{45}B^{2}ECHMPr{Re}^{2}S\eta^{10}+\frac{1}{28}A^{2}B^{2}HPr{Re}^{2}SY\eta^{8}-\frac{4}{45}A^{2}BECHPrReS\eta^{10}$$

$$-\frac{1}{45}ABECMPr^{2}ReY\eta^{10}-\frac{5}{56}AB^{2}ECHM^{2}PrRe\eta^{8}+\frac{3}{7}ABECHMPrR{e\eta }^{8}+\frac{1}{6}A^{3}B^{2}HMPrReY\eta^{6}-$$

$$110A^{3}Y\eta^{2}PrHReB-\frac{7}{3}A^{3}BPr^{2}ReY^{2}\eta^{6}+\frac{55}{6}A^{3}BPr^{2}ReY^{2}\eta^{4}-25A^{3}BPr^{2}R{eY}^{2}\eta^{2}+\frac{95}{3}A^{3}BHPrReY\eta^{4}$$

$$+\frac{4}{3}A^{3}BEcHMPr\eta^{6}-\frac{5}{3}A^{3}BEcHMPr\eta^{4}+\frac{5}{3}ABEcHMPr\eta^{4}-\frac{3}{7}PrRe\eta^{8}SHBEc-20PrEcA\eta^{2}H$$

$$+\frac{5}{28}A^{3}BPr^{2}ReY^{2}\eta^{8}-2ABECHMPrRe\eta^{6}-11ABECMPr^{2}ReY\eta^{2}+\frac{20}{3}ABECHMPrRe\eta^{4}-$$

$$\frac{5}{12}AB^{2}EcHM^{2}PrRe\eta^{4}-\frac{12}{5}A^{2}BEcHPrReS\eta^{6}+\frac{1}{3}AB^{2}EcHM^{2}PrRe\eta^{6}-\frac{8}{3}BEcHPrReS\eta^{4}$$

$$+\frac{6}{7}A^{2}BEcHPrReS\eta^{8}+\frac{2}{3}A^{2}B^{2}HPr{Re}^{2}SY\eta^{4}-\frac{4}{3}ABECMPr^{2}ReY\eta^{6}\frac{5}{12}A^{3}B^{2}HMPrReY\eta^{4}$$

$$-\frac{5}{14}A^{3}BEcHMPr\eta^{2}-\frac{16}{3}A^{3}BHPrReY\eta^{6}-\frac{4}{3}ABECHMPr\eta^{6}-\frac{4}{45}A^{3}ECPr^{2}Y\eta^{10}+\frac{12}{7}A^{3}ECHPr\eta^{8}$$

$$-\frac{5}{14}AEcPr^{2}Y\eta^{8}+20A^{3}BH^{2}Re\eta^{4}+\frac{20}{3}AEcHPr\eta^{6}+\frac{6}{7}A^{3}EcPr^{2}Y\eta^{8}-\frac{16}{3}A^{3}EcPr^{2}Y\eta^{6}$$

$$+\frac{52}{3}A^{3}EcPr^{2}Y\eta^{4}+\frac{2}{3}AEcPr^{2}Y\eta^{6}-44A^{3}EcPr^{2}Y\eta^{2}+\frac{5}{3}AEcPr^{2}Y\eta^{4}-10AEcPr^{2}Y\eta^{2})$$

$$-652B^{2}EcHMPr{Re}^{2}S+1740A^{3}BEcHMPr+210840A^{3}BHPrReY-2608A^{2}BEcHPrReS+$$

$$19676ABEcMPr^{2}ReY-652B^{2}EcHMPr{Re}^{2}S+1740A^{3}BEcHMPr+210840A^{3}BHPrReY-$$

$$2608A^{2}BEcHPrReS+19676ABEcMPr^{2}ReY+20220AEcPr^{2}Y+50400AEcHP$$ (32)

$$g=\frac{1}{75600BH^{2}Re}(450(\eta +1)A^{2}Y(2Y(\eta^{6}-\frac{181}{15}\eta^{4}+\frac{589}{15}\eta^{2}-\frac{1511}{15})B-$$

$$\frac{112(\eta^{8}-\frac{121}{14}\eta^{6}+\frac{719}{14}\eta^{4}-\frac{2011}{14}\eta^{2}+\frac{4919}{14})Ec}{225})A^{2}$$

$$-\frac{28(MRe(\eta^{8}-\frac{121}{14}\eta^{6}+\frac{719}{14}\eta^{4}-\frac{2011}{14}\eta^{2}+\frac{4919}{14})B+\frac{225\eta^{6}}{14}-\frac{195\eta^{4}}{14}-\frac{1245\eta^{2}}{14}+\frac{5055}{14})Ec}{225})$$

$$(\eta -1)Pr^{2}+420(\eta )+1)H(MReY)(\eta^{4}-\frac{3}{2}\eta^{2}-\frac{3}{2})B^{2}+(-\frac{15M\eta^{6}Ec}{7}+(\frac{41MEc}{7}-32ReY)\eta^{4}$$

$$+(-\frac{29MEc}{7}+158ReY)\eta^{2}-\frac{29MEc}{7}-502ReY)B+\frac{72(\eta^{6}-\frac{11}{3}\eta^{4}+\frac{107}{9}\eta^{2}-\frac{355}{9})Ec}{7})A^{4}-502ReY)B$$

$$+\frac{3BS(ReY(\eta^{6}-\frac{25}{3}\eta^{4}+\frac{31}{3}\eta^{2}+\frac{31}{3})B-\frac{112(\eta^{8}-\frac{121}{14}\eta^{6}+\frac{257}{14}\eta^{4}-\frac{163}{14}\eta^{2}-\frac{163}{14})Ec}{45})ReA^{3}}{14}+$$

$$+(-\frac{15M^{2}(\eta^{6}-\frac{41}{15}\eta^{4}+\frac{29}{15}\eta^{2}+\frac{29}{15})EcR{eB}^{2}}{28}+(\frac{18MRe\eta^{6}Ec}{7}-\frac{66M(Re+\frac{28}{33})Ec\eta^{4}}{7}+$$

$$((\frac{214}{7}MRe+2M)Ec+30ReY)\eta^{2}+(2M-\frac{710}{7}MRe)Ec-150ReY)B+16Ec(\eta^{4}+6\eta^{2}-24))A^{2}$$

$$-\frac{2BS(MRe(\eta^{8}-\frac{121}{14}\eta^{6}+\frac{257}{14}\eta^{4}-\frac{163}{14}\eta^{2}-\frac{163}{14})B+\frac{135\eta^{6}}{7}-\frac{705\eta^{4}}{7}+\frac{135\eta^{2}}{7}+\frac{135}{7})EcReA}{15}-6(MRe(\eta^{4}$$

$$-4\eta^{2})+11)B+10\eta^{2}+10)Ec((\eta -1)Pr+50400B(\frac{3}{2}+(\eta^{4}-6\eta^{2}+5)A^{4}+(-3\eta^{2}+3)A^{2})H^{2}Re)$$ (33)

## 4. Result and discussion

**Fig 1:**
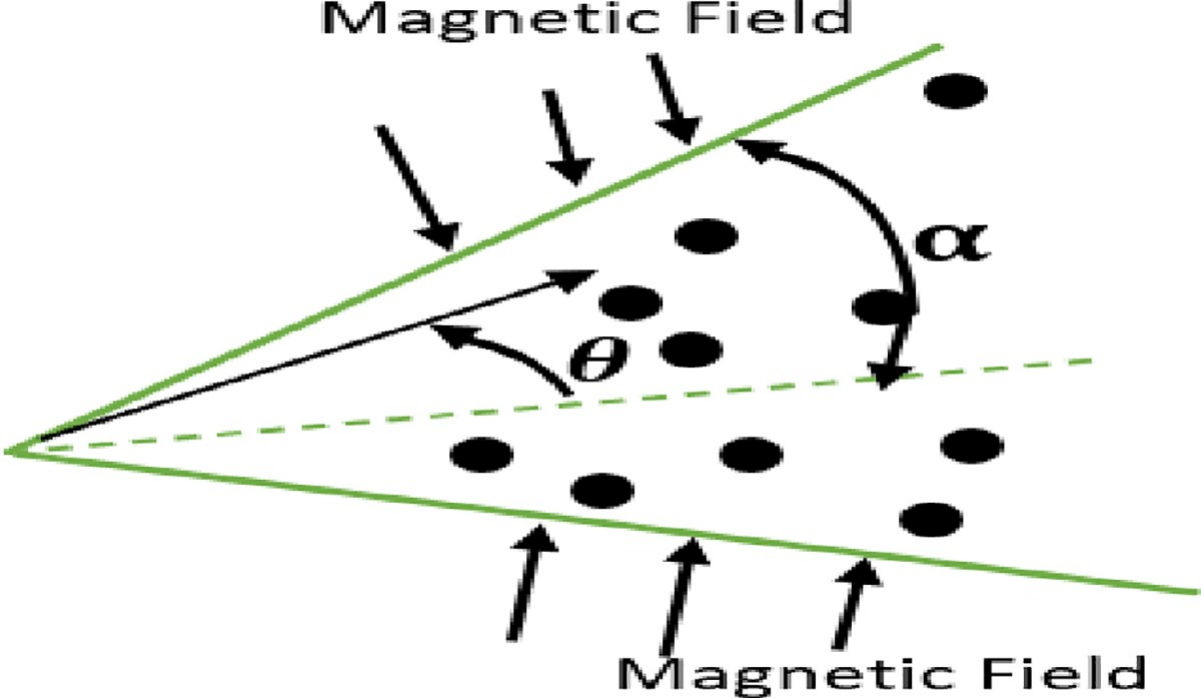
Geometry of the problem.

**Fig 2:**
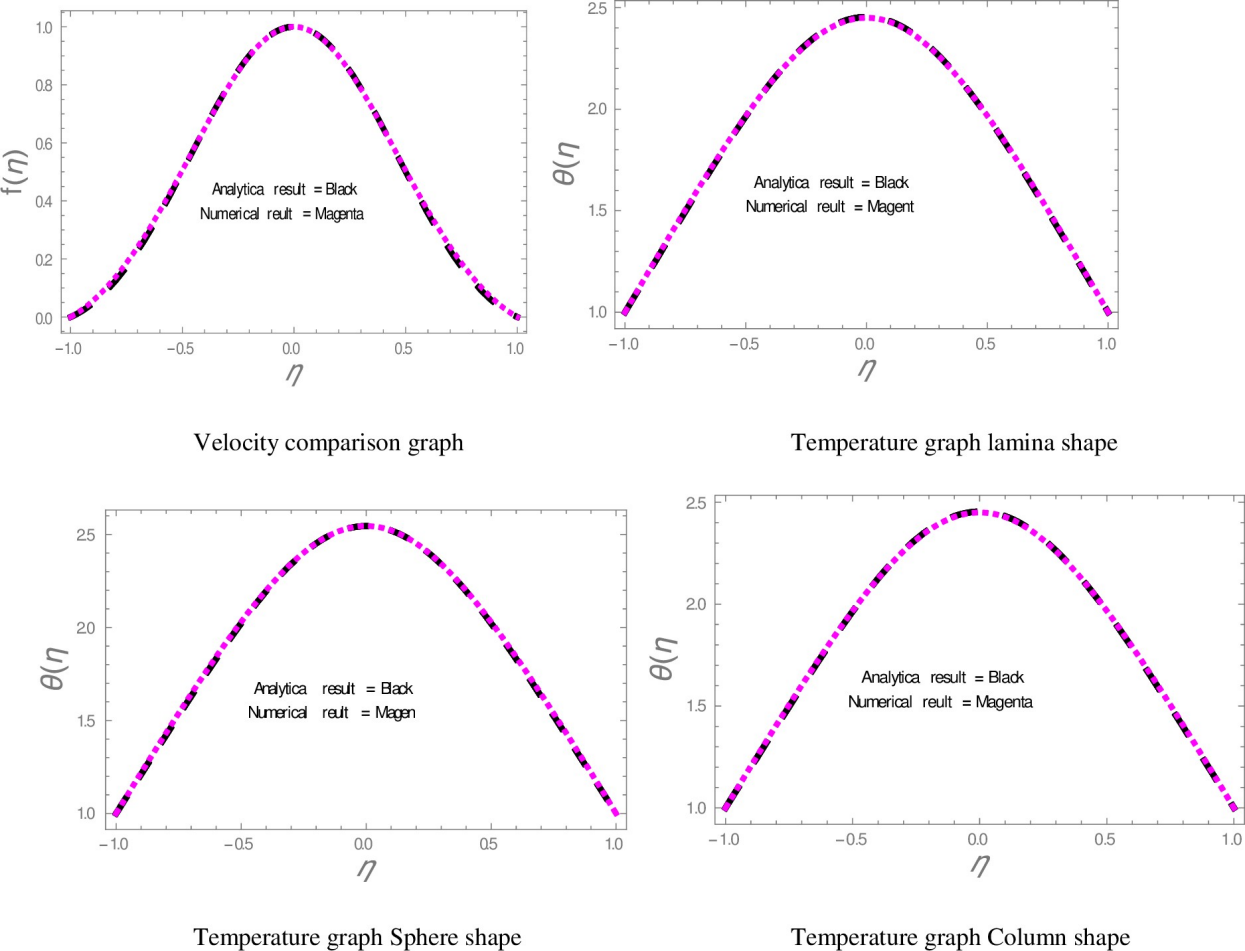
Comparison of results in diverging channel.

**Fig 3:**
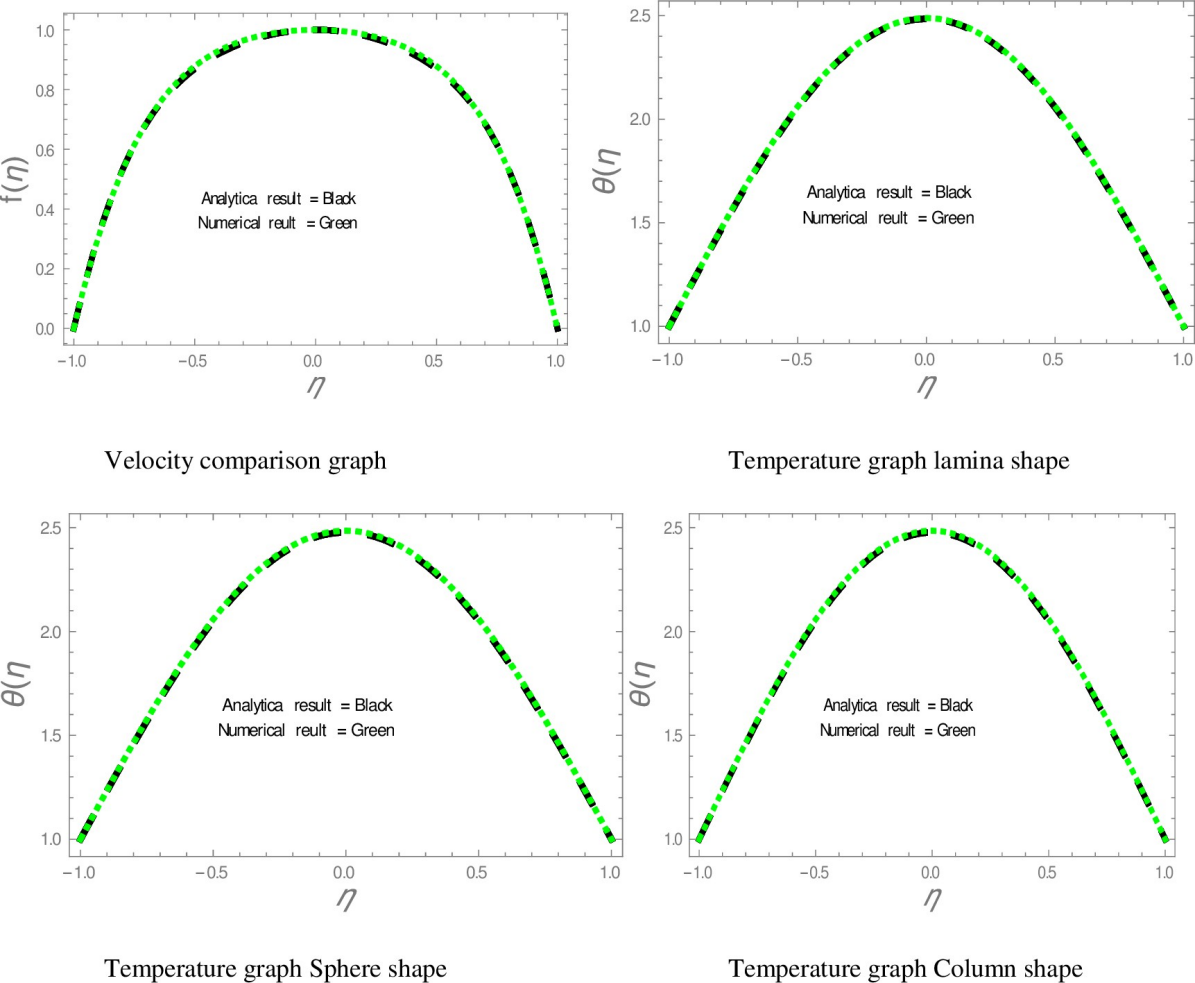
Comparison of results in converging channel.

**Fig 4:**
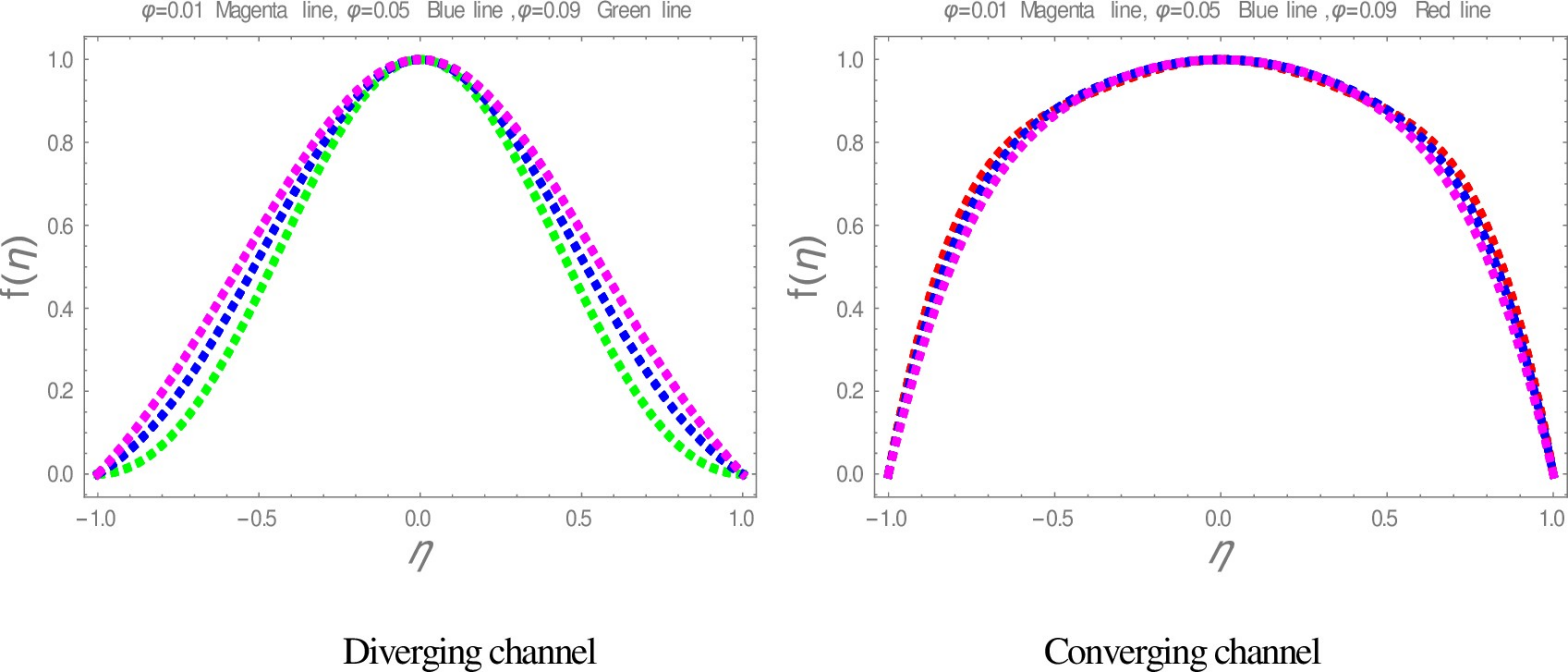
The effect of *ϕ* on velocity graphs.

**Fig 5:**
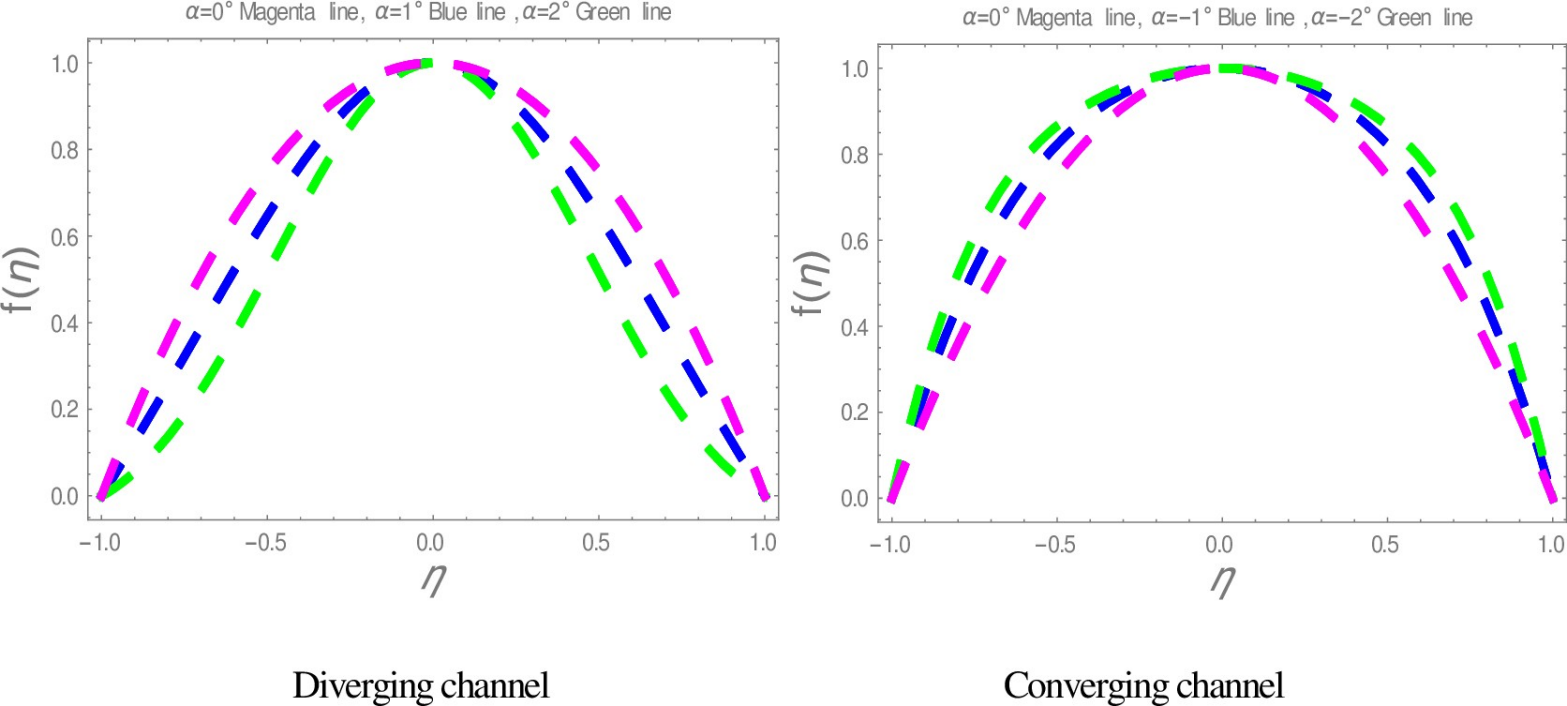
The effect of *α* on velocity graphs.

In this section, nanofluid flow and heat transfer between divergent-convergent channels with nanoparticles shapes effects are discussed. Also, the results obtained by using Homotopy perturbation are clarified with the numerical NDsolve method. Geometry of the problem is presented in Fig 1. The results of the comparative solutions are shown in Figs 2 and 3. Fig 4 displays the variation in the velocity of ZnO-water nanofluid within divergent-convergent channels under the influences of *ϕ*. From Fig 4, it is also noted that the velocity of nanofluid decreases with increase *ϕ* in a divergent channel but an opposite behaviour is found in the convergent channel. The effects of *α* on the velocity of nanofluid are shown in Fig 5. It is also observed from Fig 5 that the velocity of nanofluid has an inverse relation within the divergent channel but direct relation within the convergent channel.

**Fig 6:**
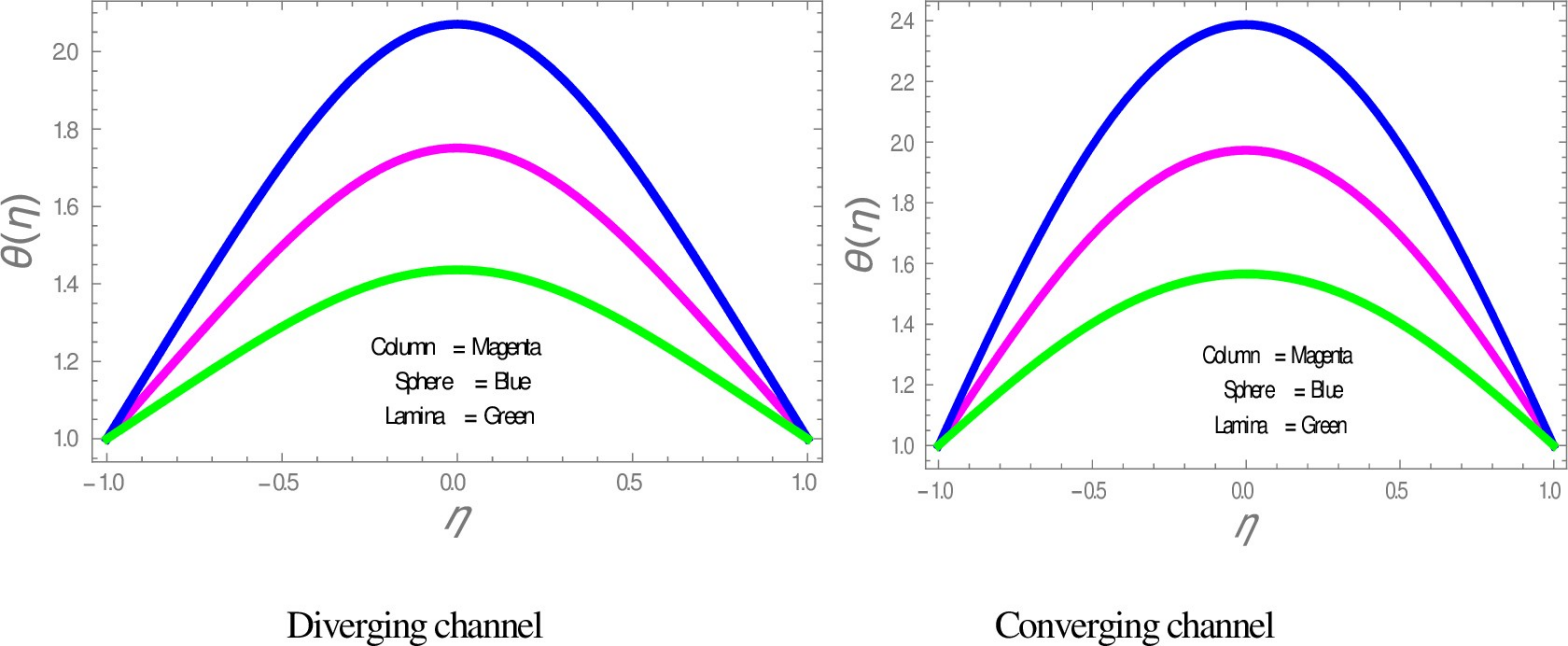
The effect of particles on temperature profiles.

**Fig 7:**
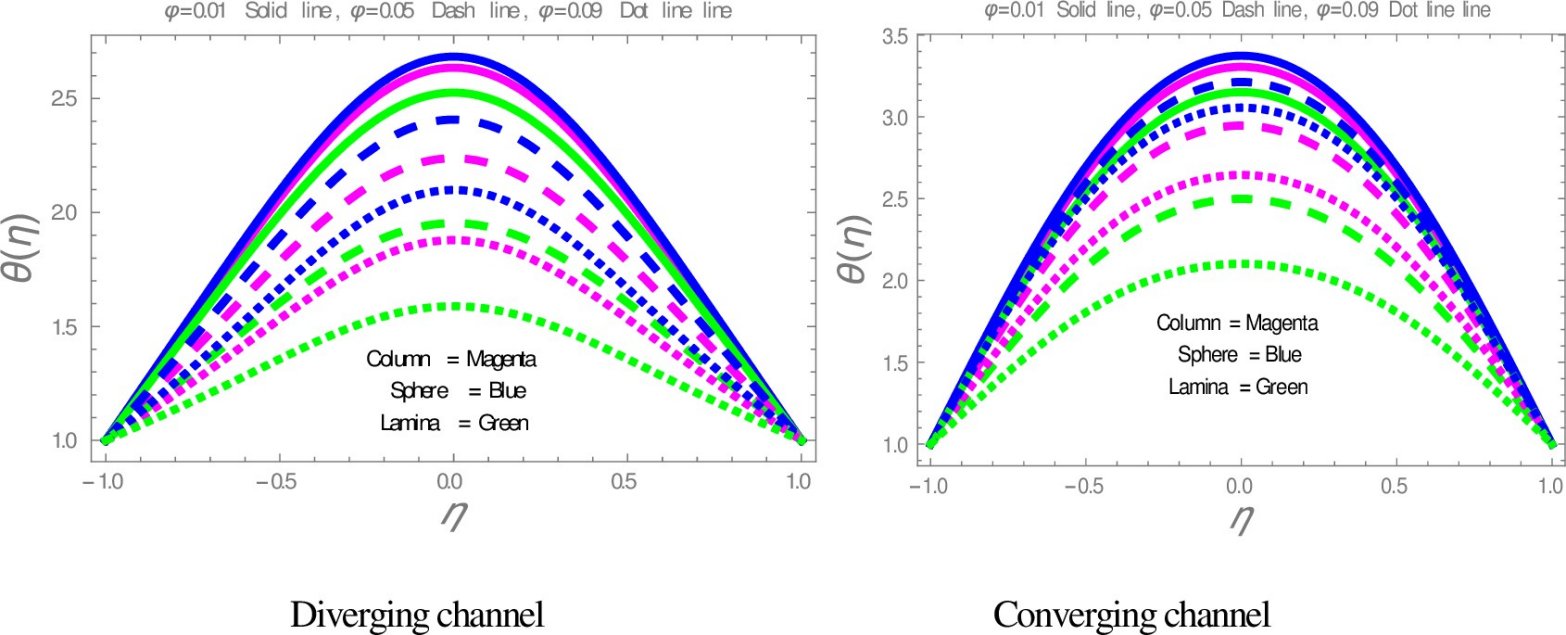
The effect of *ϕ* on the temperature profile.

**Fig 8:**
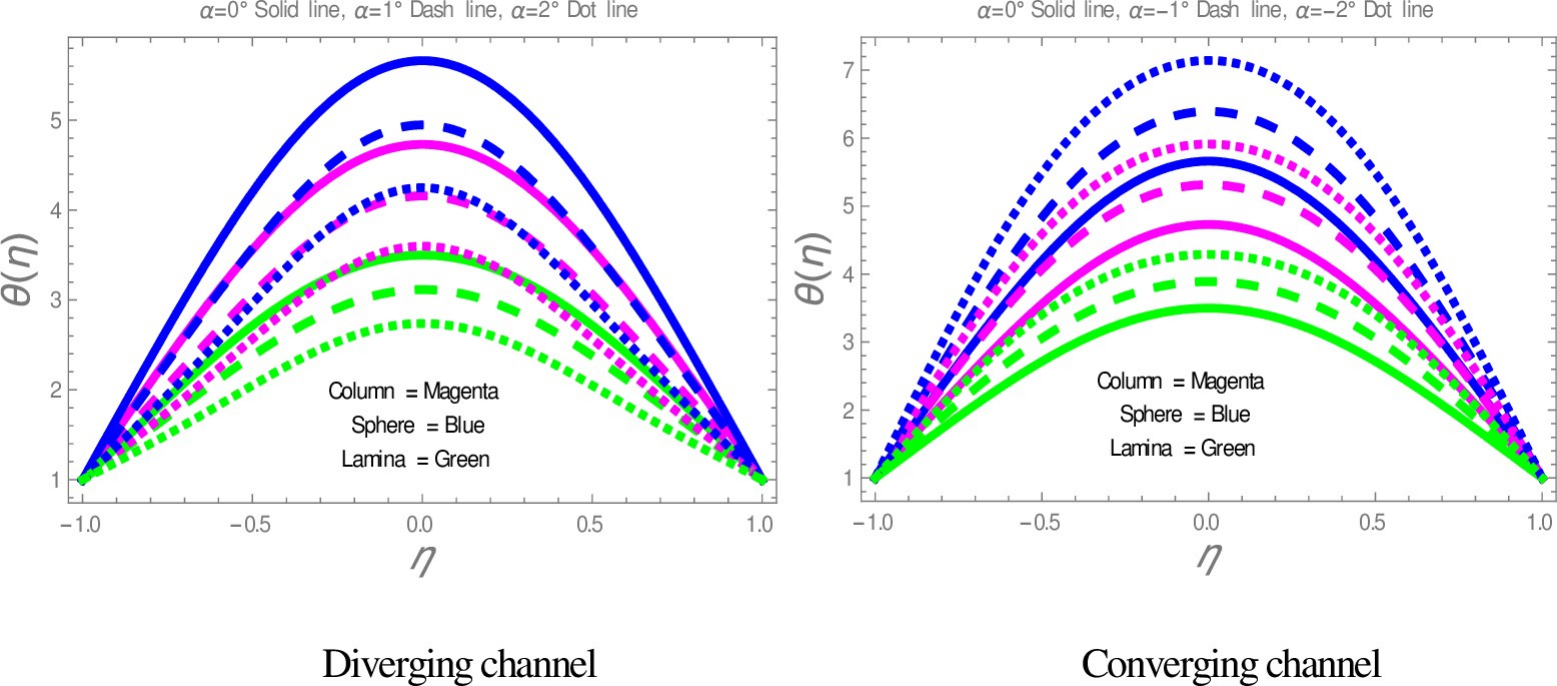
The effect of *α* on temperature profiles.

The effect of shape factor on the temperature within divergent-convergent channels is presented in Fig 6. Fig 6 illustrates that sphere shape nanoparticles have higher, whereas lamina shape nanoparticles have lower performance in temperature disturbance within divergent-convergent channels. Fig 7 presents the variation in temperature profile with the influences of *ϕ*. It is observed in Fig 7 that the temperature of nanofluid is a decreasing function of solid volume fraction within divergent-convergent channels. It is also noted from Fig 7 that the performance of sphere shape nanoparticles in temperature profile is higher than column and lamina nanoparticles within divergent-convergent channels. Fig 8 depicts the behaviors of *α* in the temperature profile. The temperature of nanofluid has a decreasing function of *α* in the divergent channel, while an opposite result has found in the convergent channel.

**Fig 9:**
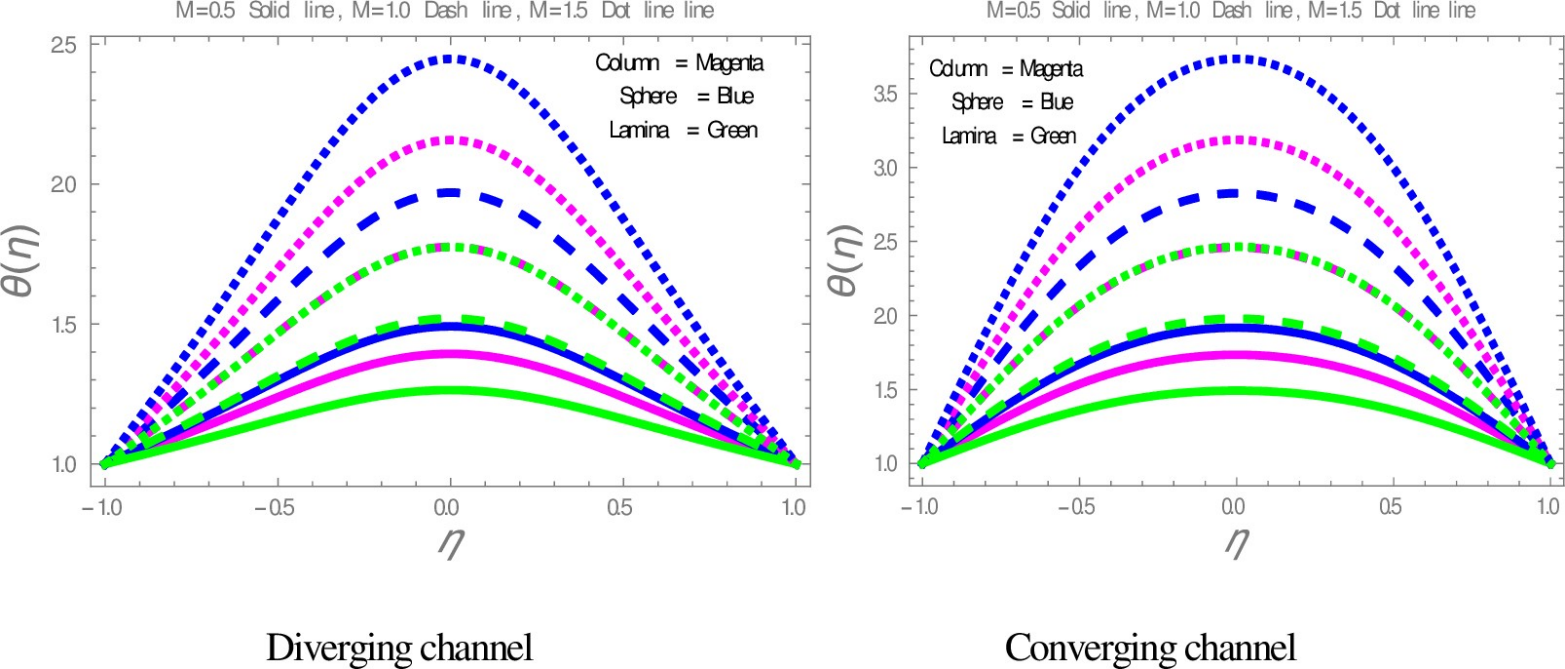
The effect of *M* on temperature profiles.

**Fig 10:**
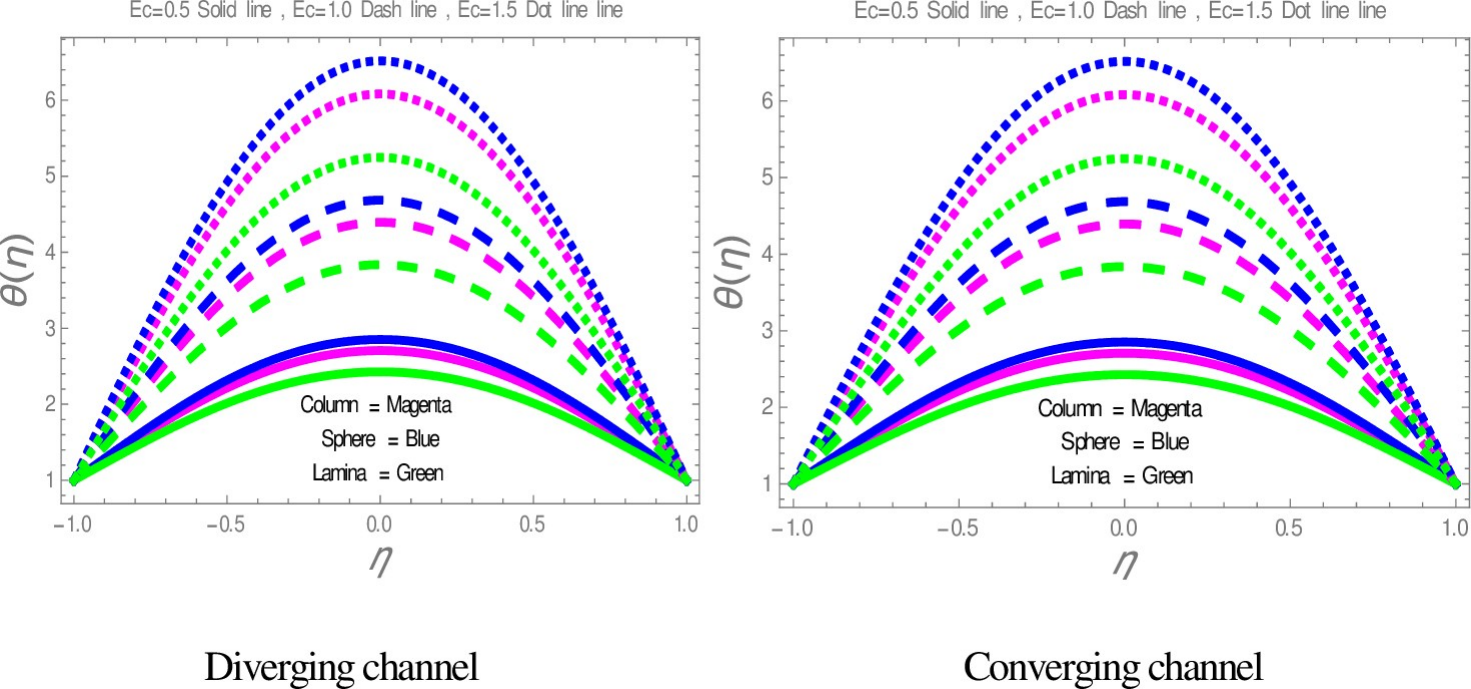
The effect of *Ec* on temperature profiles.

**Fig 11:**
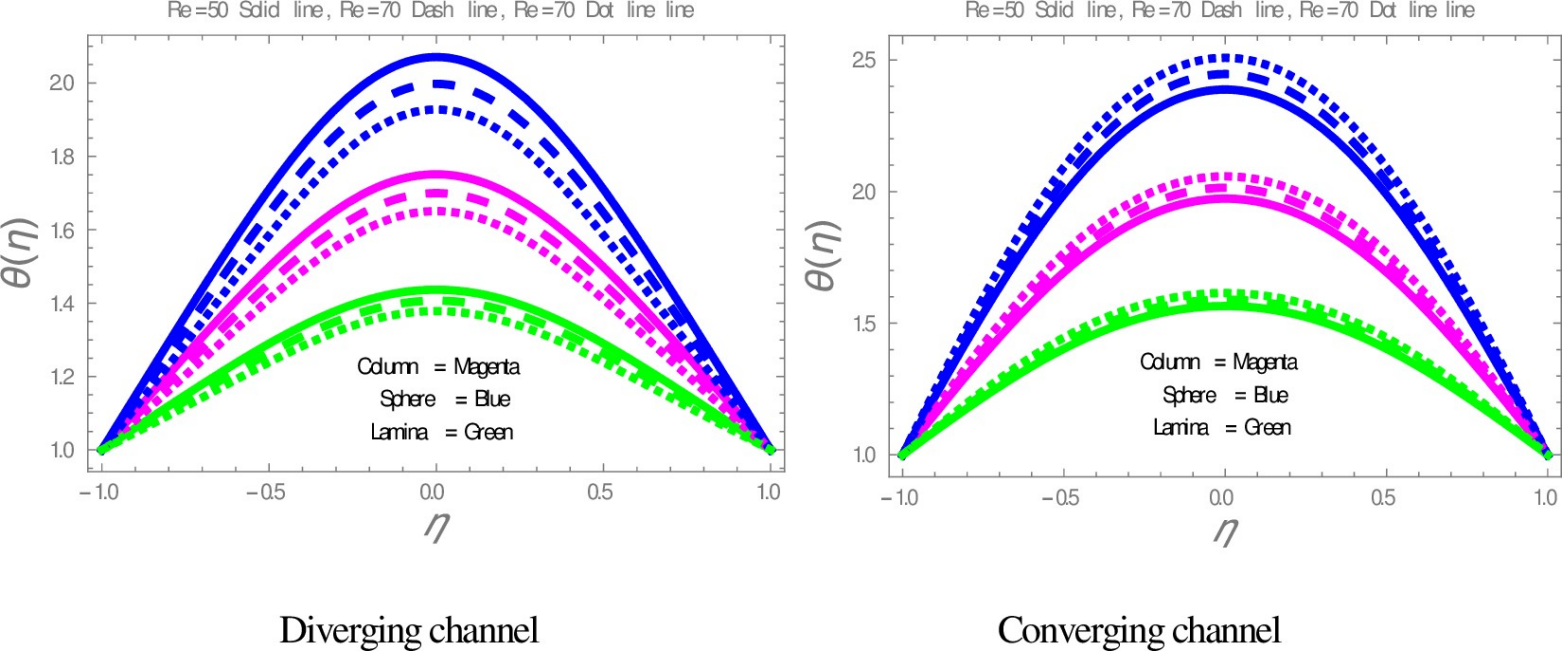
The effect Re on temperature profiles.

**Fig 12:**
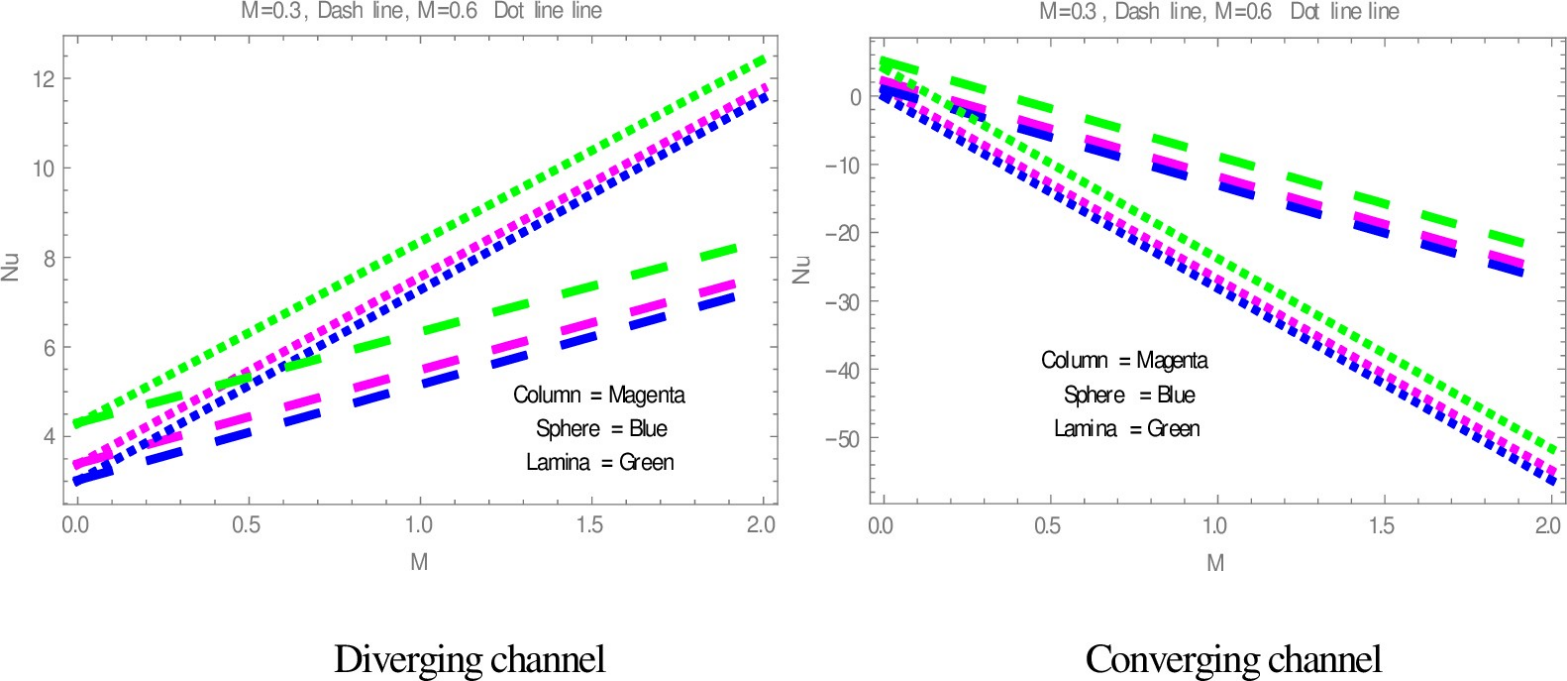
The effect of *M* on heat transfer.

**Fig 13:**
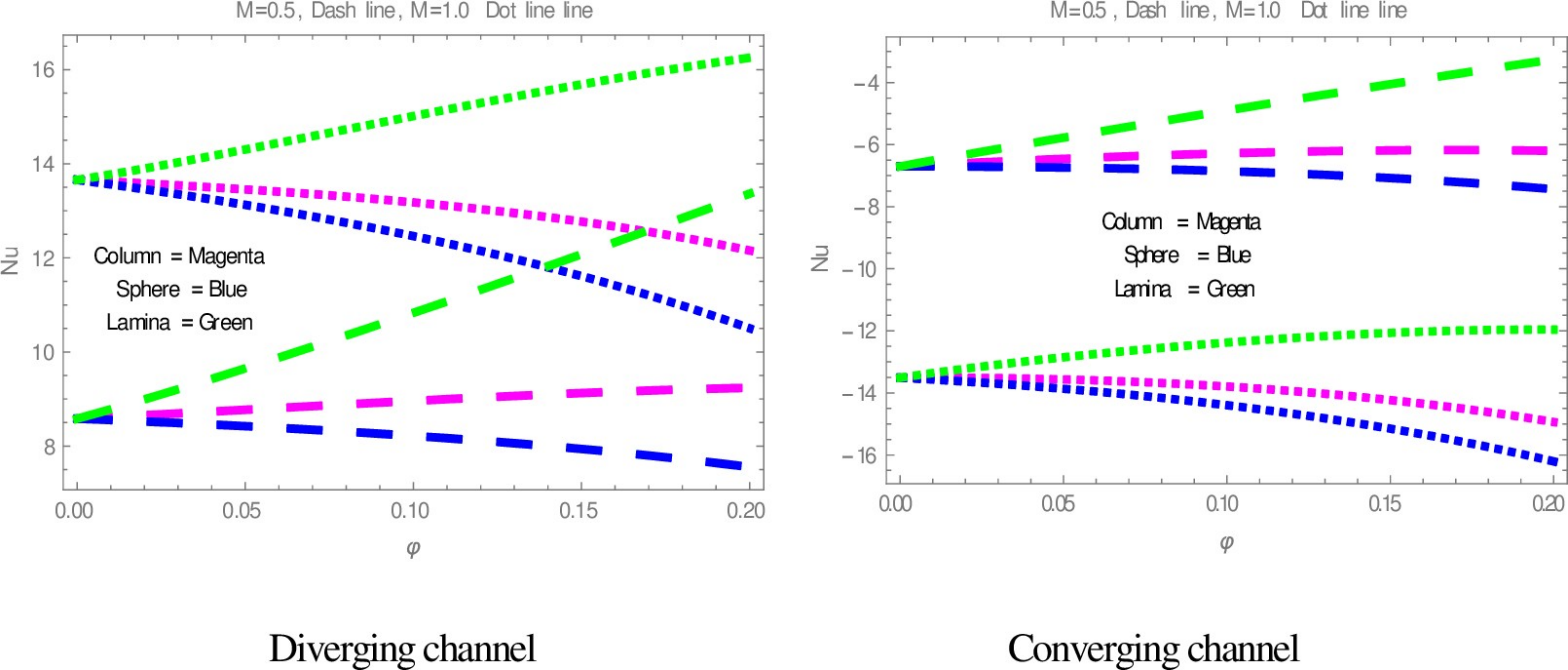
The effect of *ϕ* on heat transfer.

Fig 9 is plotted to check the effect of *M* on temperature profile. The figure illustrates that the temperature of nanofluid within divergent-convergent channels has a direct relation with *M*. Fig 10 shows the effect of *Ec* on temperature profile within divergent-convergent channels. Form Fig 10, it is noted that the effect of *Ec* within divergent channel is similar to the effect of *Ec* in convergent channel. It is also noted from Figs 9, 10 performance of sphere shape nanoparticles is lower than other shapes of nanoparticles in divergent-convergent channels with the effect of *M* and *Ec*. Variation in temperature profile with the influences of Re is presented in Fig 11. It is analyzed from Fig 11, Re has an inverse and direct relation with temperature distribution within the divergent channel and convergent channel, respectively. The performance of nanoparticles is similarly examined with the impacts of *M* and *Ec*. Figs 12, 13 are sketched to investigate the heat transfer rate. From Figs 12, 13, we observe that the heat transfer rate in lamina shape nanoparticles is better than other nanoparticles shapes. The comparison of homotopy perturbation method and NDsolve solutions are presented in Table 3. Table 4 Shows comparison of solution with already published paper.

**Table 3:** Comparison of values for *ϕ* = 0.2, *M* = 1.0, *R*_*e*_ = 2.0 and *α* = 0.

*x*	*f*(*x*)	*f*(*x*)	*f*′(*x*)	*f*′(*x*)
	ND	HPM	ND	HPM
1	0.9900	0.9900	-0.2000	-0.2000
2	0.9600	0.9600	-0.4000	-0.4000
3	0.9099	0.9099	-0.6000	-0.6000
4	0.8400	0.8400	-0.8000	-0.8000
5	0.7500	0.7500	-1.0000	-1.0000
6	0.6400	0.6400	-1.2000	-1.2000
7	0.5100	0.5100	-1.4000	-1.4000
8	0.3600	0.3600	-1.6000	-1.6000

**Table 4:** Comparison of results with already published work [27].

	Convergent channel	Convergent channel	Diverging channel	Diverging channel
Re	Abbasbandy and Shivanian Results	Present Results (HPM)	Abbasbandy and Shivanian Results	Present Results (HPM)
10	1.7845468	1.7848768	2.2519486	2.2515638
20	1.5881535	1.5882108	2.5271922	2.5243141
30	1.4136920	1.4139204	2.8326293	2.8332759
40	1.2589939	1.2595367	3.1697121	3.1720138
50	1.219890	1.1229166	3.5394156	3.5456609
60	1.0007429	1.0019168	3.9421402	3.9563603
80	0.7985672	0.7982056	4.8450718	4.8974887

## 5. Conclusion

The effects of nanoparticle shapes (sphere, column and lamina) in a dynamic of water conveying Zinc oxide through divergent-convergent channels are computed by using analytical and numerical techniques. The effect of the magnetic field and joule dissipation are also examined. A fixed value of Pr = 6.2 is used in this study. Apropos to the above discussion, the following dedications are pointed below:

- The sphere shape nanoparticles have a greater role in temperature distribution and the lowest role in heat transfer.
- The lamina shape nanoparticles have a greater role in heat transfer and the lowest role in temperature distribution
- The column shape nanoparticles have a middle role in heat transfer and temperature distribution.

## Abbreviations

T: Temperature of nanofluid(k)
K: Thermal conductivity (W/m.K)
Cp: Specific heat capacity (J/kg K)
Ρ: Density (kg/m^3^)
ϕ: Solid volume fraction
μ: Dynamic viscosity (N.sm^-2^)
*σ**: Stefan Boltzmann constant (w.m^-2^. k^-4^)
*k**: Absorption coefficient (m^-1^)
p: pressure
v_r_: radial velocity
$\alpha_{n_{f}}$: Thermal diffusivity of the nanofluid (m/s^2^)
m: Shape variable
n_f_: Nanofluid
f: Liquid
s: Solid
Ec: Eckert number
M: Hartmann number
Pr: Prandtl number
Re: Reynolds number
α: Divergent-Convergent parameter
B: Electromagnetic induction
σ: Fluid conductivity
